# Development of SNP for *Sebastes* Species Identification With Special Focus on the Cryptic Species Complex of *Sebastes norvegicus*

**DOI:** 10.1002/ece3.70767

**Published:** 2025-01-08

**Authors:** Torild Johansen, Tanja Hanebrekke, Francois Besnier, Jon Ivar Westgaard, Ingrid Marie Bruvold, Geir Dahle, Laila Unneland, Helle Torp Christensen, Atal Saha

**Affiliations:** ^1^ Institute of Marine Research (IMR) Tromsø Norway; ^2^ Institute of Marine Research (IMR) Bergen Norway; ^3^ Greenland Institute of Natural Resources Nuuk Greenland; ^4^ Centre for Coastal Research, Department of Natural Sciences University of Agder Kristiansand Norway

**Keywords:** discriminatory SNPs, golden redfish, management advice, *S. marinus*

## Abstract

The genus *Sebastes* in the North Atlantic comprises of long lived deep‐waters species that have been extensively fished upon, and many stocks are severely depleted across the Atlantic. This is particularly evident for the species *Sebastes norvegicus* . In recent papers, cryptic species have been indicated within this genus and molecular markers are therefore needed to provide identification for the *Sebastes* species, including the cryptic species as a basis for advice regarding management and rebuilding of the stocks. A suite of 2800 Single Nucleotide Polymorphism (SNP) markers were identified from ddRAD sequencing data, of which 56 SNPs were organized in two multiplex reactions and tested on 191 *Sebastes* spp. from different sampling locations from Norway and Greenland. Good‐quality amplification products were successfully obtained from 49 SNP markers for *Sebastes* species ID, and 3 TaqMan probes were designed to successfully assign *S. mentella* , *S. viviparus*, and the two cryptic species *S. norvegicus* types A and B. A total 47 SNPs were biallelic, with averaged *H*_E_ per locus ranging between 0.053 and 0.50. This SNP‐based method establishes a foundation for genetically identifying the Northeast Atlantic *Sebastes* species. The findings presented should be followed by an effort to look for morphological characters to recognize the *S. norvegicus* cryptic species on site. In general, the SNP markers are a proper tool for monitoring the distribution of the species from a management perspective.

## Graphical abstract

New SNP markers to ID species of genus *Sebastes* in the North Atlantic.

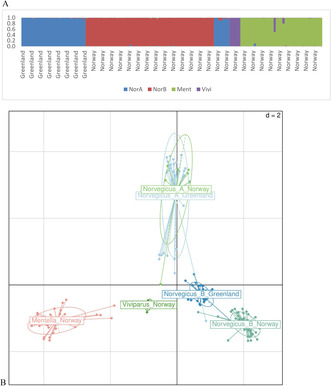

## 1 Introduction

The genus *Sebastes* in the Northeast Atlantic is composed of three species. This includes the *Sebastes mentella* , *S. viviparus* , and *S. norvegicus* (“golden redfish,” formerly *S. marinus* ). The golden redfish is the largest species of the three and is found across the North Atlantic along with *S. mentella* (Barsukov, Litvinenko, and Serebryakov 1985). Golden redfish is found at 200–800 m depth (Barsukov, Litvinenko, and Serebryakov 1985) depending on the area and can be distinguished from *S. mentella* by the golden color and smaller eyes in adult specimens (Power and Ni 1985). However, in recent years the golden redfish stock collapsed in the Norwegian fisheries and a zero catch advice was introduced in 2015 (Bakketeig et al. 2015), and similar problems are observed also in other areas (ICES 2024, Brûlé et al. 2024). *S. viviparus* is the smallest of the three and in some areas caught in the same trawl, but not of any commercial interest.

In a recent genetic study, two possible cryptic species were indicated within golden redfish Saha, Johansen, et al. (2017). Saha, Hauser, et al. (2017) suggested two genetically assigned cryptic species in Greenland and Norwegian waters that was denoted *S. norvegicus* A and B. The morphological characters for *S. norvegicus* A and B were unknown at the time (Saha, Johansen, et al. 2017; but see Schmidt and Trautner 2005). Both *S. norvegicus* A and B have been genetically identified by microsatellite genotyping (Saha, Hauser, et al. 2017). For management purposes, it is important to have a robust method for species identification among the genus *Sebastes*. Therefore, genetic markers that diagnostically can distinguish between Northeast Atlantic redfish species are needed for proper monitoring of the *Sebastes* complex. Cryptic speciation is not an uncommon process in marine environments (Bickford et al. 2007; Miglietta, Faucci, and Santini 2011), where species can diversify without necessarily undergoing associated morphological change. The apparent external similarities in sister taxa can be caused by recent reproductive isolation or stabilizing selective pressures resulting in morphological stasis (see Fišer, Robinson, and Malard 2018 and references therein). This can cause new species to remain undetected for a long time, as taxonomy has been mainly reliant on external morphology in the past. However, accurate information on diversity among commercially important species is especially important to maintain and adjust sustainable management strategies for avoiding excessive harvest. In general, for deep‐sea species, it can be particularly challenging to capture the range of variation across spatial and temporal gradients. Genomic analyses provide an opportunity to detect such variation on a genetic level, which has facilitated the discovery of spatial genetic differentiation and cryptic speciation in marine environments (Miglietta, Faucci, and Santini 2011). Several novel species have been described in recent years within the genus *Sebastes* using genetic methods, including mitochondrial DNA (Rocha‐Olivares, Rosenblatt, and Vetter 1999), amplified fragment length polymorphisms (Kai, Nakayama, and Nakabo 2002), and microsatellites (Hyde et al. 2008; Saha, Johansen, et al. 2017).

The present project aims to identify SNP markers that can easily distinguish between the Northeast Atlantic *Sebastes* species including the cryptic *S. norvegicus* A and B for monitoring purposes for a sustainable fishery for this genus.

## 2 Methods

Genomic DNA was extracted from gills filaments preserved in ethanol by using the E‐Z 96Tissue DNA kit, following manufactures protocol Omega Bio‐Teck. Inc. Norcross GA, USA) from 506 *Sebastes* specimens from previous projects (e.g., Saha, Johansen, et al. 2017, Saha et al. 2021): 174 *Sebastes norvegicus* , 277 *S. mentella* , 35 *S. viviparus* and 20 *S. fasciatus* specimens (only known in the Northwest Atlantic) were collected and morphologically identified from eight locations across the North Atlantic. The two *S. norvegicus* types (A and B) have mainly been assigned by microsatellites (Saha, Hauser, et al. 2017), except for the samples from 2014 that indicated new morphological characters differentiating the two types (Ingrid Brudvold personal communication).

To identify the SNP markers we revisited the ddRAD sequences from Saha et al. 2021. The ddRAD libraries were prepared for sequencing according to the protocol by (Peterson et al. 2012) using MspI and EcoRI restriction enzymes (NEB, USA)). After preparation of the library, sequencing was performed using a MiSeq (Illumina, California, USA) and 500 cycles MiSeq Reagent Kit v2 (for more details on the ddRAD sequencing conditions see Saha et al. 2021).

Reads were aligned to the *Sebastes* assembly (Malmstrøm et al. 2016) using mem algorithm from bwa‐mem V2 (Vasimuddin et al. 2019), and SNP calling was done with Samtools V1.8 (Li et al. 2009) and bcftools (Li 2011). SNPs were then filtered according to Phred‐scaled quality score, keeping only markers with quality score greater than 500. PCA plot of the ddRAD SNP are found in (Figure S1). Diagnostic SNPs were then selected between each sample pair‐wise ( *S. norvegicus* A; *S. norvegicus* B; *S. mentella and S. viviparus*). For a given pair of samples, SNPs with coverage larger than 20× in both samples, and homozygous for alternative alleles between samples were considered as candidates for diagnostic markers. Finally, one SNP per contigs greater than 20 kb was retained to minimize linkage disequilibrium.

**FIGURE 1:**
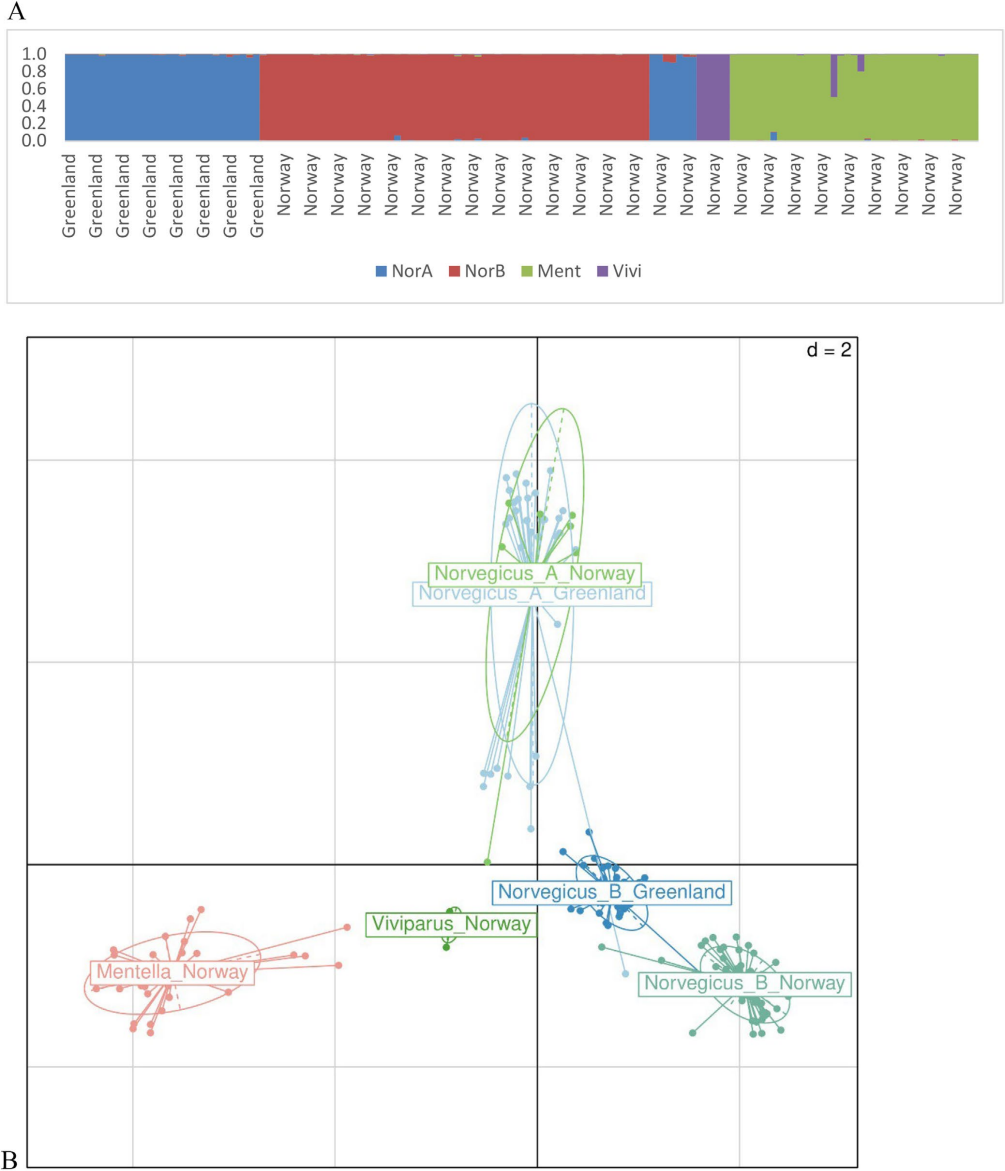
Structure plot of the *Sebastes* species (See text for details) (A) and (B) PCA plot based on the 47 SNP markers for the 191 *Sebastes* sp. Here, PC1 & PC2 explain 40.64% & 27.72% of variances, respectively.

The large panel of SNPs detected from the ddRAD sequencing were run through the Assay Designer v 4.0.0.2 (MassArray Typer Version 4.1; Agena Bioscience, Gabriel, Ziaugra, and Tabbaa 2009). Each run produced numerous assays, but after close inspection and careful review two of the thirty assays (i.e., 29 and 27 SNP loci) were chosen manually as the best discriminatory combinations of loci between the species in question including *S. norvegicus* A and B. The panel of 56 SNPs were then pooled into two multiplex reactions and 191 individuals (Table 1) were analyzed on a MALDI‐TOF instrument (Agena Bioscience) and genotyped with the Typer 4.0 software (Agena Bioscience). Based on the results from the 56 SNPs, three SNPs were selected for a fast and robust identification of *Sebastes* species for monitoring purposes using TaqMan probes. These were subsequently analyzed by a three TaqMan SNP Genotyping assays using 1× Perfecta qPCR fast mix (Quantabio, Massachusetts, USA), 0.5—1× TaqMan SNP Genotyping assay (ThermoFisher, USA), 20–50 ng DNA and adding dH_2_O to a final volume of 5 μL. The assays were analyzed on a QuantStudio 6 real‐time PCR instrument (Thermo Fisher Scientific, Waltham, USA) with an initial denaturation of 95°C for 10 min followed by 40 cycles of 95°C for 15 s, 60°C for 1 min.

**TABLE 1:** The sample composition of the current study.

Location	Species	Code	*N*	Time	Lat/Long (Mean)	Depth (m)	Age (years)	Length (cm)
East Greenland	*S. norvegicus*	NorvBGr*	20	Feb, 2011	64.26/−35.15	365–370 (367)		26–57 (38)
		NorvBGr*	20	Aug, 2011	62.2/−40.65	188–332 (239)		17–49 (28)
		NorvAGr*	39					
Norway	*S. mentella*	NorvBNo*	57	Oct, 2001	69.19/15.08	195–417		29–54 (36)
		NorvANo*	2					
		NorvBNo	2	May, 2014	65–68*N*/	374–379	26–36	
		NorvANo	6			352–374	0.23–34	
		MenNo*	18	Nov, 2011	69.38/15.14	423–512 (453)		20–40 (30)
	*S. viviparus*		12	May 2014	65–71/	477–625	0.17–39	
			10	Nov, 2016		367–377 (372)		28–63 (36)
		ViviNo	5	May, 2014	70.5/20.52	374–468	0.11–32	
		Total	191					

* The redfish were previously assigned to species by microsatellites as in Saha, Johansen, et al. 2017 and part of the ddRAD. Total 191 redfish were tested for both SNPsets containing 49 and three loci (the Taqman assay).

Observed (*H*_O_) and expected heterozygosity (*H*_E_) as well as the inbreeding coefficient (*F*_IS_) were computed with GenAlEx (Peakall and Smouse 2006). The genotype distribution of each locus was compared with the expected Hardy–Weinberg distribution (HWE) as was the departure from Linkage Disequilibrium (LD), using the program GENEPOP 7 (Rousset 2008).

To test for the neutrality of the identified loci, island models allowing differences in the population sizes (Foll and Gaggiotti 2008) were applied. The tag sequences containing outlier SNPs were blasted in the NCBI database using the “BLASTN” window to explore their possible association with any functional parts of the genome. Both model‐based (Pritchard, Stephens, and Donnelly 2000) and non‐model‐based (Jombart, Devillard, and Balloux 2010) clustering approaches were used for the clustering of individuals in the study.

An unrooted Neighbor‐Joining tree was drawn to visualize the segregation of the *Sebastes* species based on 191 individuals and 49 SNPs. The extent of divergence among *Sebastes* spp. was quantified by the chord distance (*D*_CE_, Cavalli‐Sforza and Edwards 1967). Pair‐wise distances were used to construct an unrooted‐phenogram using the Neighbor‐Joining (NJ) algorithm (Saitou and Nei 1987) available in Populations (Langella 2002). A total of 1000 bootstraps were run on loci to assess confidence of nodes in the tree. The Phylip format tree generated by Populations was viewed in FigTree 1.4.2 (http://tree.bio.ed.ac.uk/software/figtree/FigTree v1.4.2). PCA was conducted using R package adegenet v.2.1.1 (Jombart and Ahmed 2011) and for the assigning of individuals in STRUCTURE we used Burn‐in period of 300,000, 1 million Markov chain Monte Carlo (MCMC) iterations, and 10 replicates for each *K* = 1–7. Structure selector selected *K* = 4 and Clump averaged the 10 replicate for each *K* (Pritchard, Stephens, and Donnelly 2000).

## 3 Results and Discussion

A suite of 2800 SNP markers were identified from ddRAD sequencing data. In total, 30 assays were constructed of which two assays were manually selected as they showed a good combination of SNP markers for species assignment for all the relevant species. The 56 SNPs were then tested on 191 *Sebastes* spp. from different sampling locations (Table 1). Good‐quality amplification products were successfully obtained from 49 SNP markers for *Sebastes* species ID, a total of which 47 were bi‐allelic (Table 2). The observed heterozygosity (*H*_o_) ranged from 0.005 to 0.363 and expected heterozygosity (*H*_e_) ranged from 0.053 to 0.50. The values of inbreeding coefficient (*F*_IS_) were from 0.273 to 1.00. As expected, all the 47 SNPs showed deviation from HWE but none of these loci were supported to be under selection and no hits were found when blasting the sequences to Genbank (Altschul et al. 1990). The observed deviations from HWE showed heterozygote deficiencies supporting the presence of reproductively isolated groups of *Sebastes* in the sample collection. Similar observations were reported in other studies based on microsatellite marker systems (e.g., Saha, Johansen, et al. 2017; Schmidt and Trautner 2005).

**TABLE 2:** Summary table for the 47 SNP markers distributed in 2 multiplex reactions.

Locus	*N*	Na	*H*o	*H*e	*F*	Signif	MAF	A1	A2
SEB1	185	2	0.016	0.126	0.871	***	0.068	A	C
SEB2	186	2	0.005	0.057	0.906	***	0.030	C	T
SEB3	185	2	0.054	0.102	0.471	***	0.054	C	T
SEB4	185	2	0.005	0.058	0.906	***	0.030	A	C
SEB5	140	2	0.050	0.379	0.868	***	0.254	A	G
SEB6	118	2	0.085	0.387	0.781	***	0.263	A	G
SEB7	139	2	0.072	0.370	0.805	***	0.245	A	C
SEB8	175	2	0.320	0.496	0.355	***	0.457	C	T
SEB9	175	2	0.109	0.368	0.705	***	0.243	C	T
SEB10	141	2	0.035	0.415	0.915	***	0.294	A	G
SEB11	141	2	0.014	0.406	0.965	***	0.284	A	C
SEB12	168	2	0.363	0.500	0.273	***	0.485	C	T
SEB13	115	2	0.061	0.390	0.844	***	0.265	C	T
SEB14	138	2	0.101	0.405	0.750	***	0.283	G	T
SEB15	139	2	0.108	0.454	0.762	***	0.349	A	G
SEB16	134	2	0.119	0.400	0.701	***	0.276	A	T
SEB17	138	2	0.000	0.096	1.000	***	0.051	A	G
SEB18	129	2	0.217	0.467	0.535	***	0.372	A	G
SEB19	132	2	0.318	0.449	0.292	***	0.341	A	G
SEB20	137	2	0.161	0.441	0.636	***	0.328	A	G
SEB21	182	2	0.154	0.367	0.580	***	0.242	C	T
SEB22	132	2	0.356	0.499	0.286	**	0.473	A	G
SEB23	124	2	0.266	0.480	0.445	***	0.399	A	G
SEB24	142	2	0.035	0.499	0.929	***	0.475	A	G
SEB25	142	2	0.007	0.375	0.981	***	0.250	A	G
SEB26	140	2	0.121	0.399	0.695	***	0.275	C	T
SEB27	180	2	0.128	0.477	0.732	***	0.392	C	T
SEB28	175	2	0.189	0.499	0.622	***	0.477	G	T
SEB29	185	2	0.000	0.053	1.000	***	0.027	C	T
SEB30	188	2	0.005	0.057	0.906	***	0.029	C	T
SEB31	174	2	0.029	0.072	0.600	***	0.037	C	T
SEB32	184	2	0.043	0.294	0.852	***	0.179	G	T
SEB33	186	2	0.027	0.335	0.920	***	0.212	C	T
SEB34	183	2	0.098	0.500	0.803	***	0.486	A	G
SEB35	143	2	0.126	0.463	0.728	***	0.364	C	T
SEB36	184	2	0.033	0.352	0.907	***	0.228	C	T
SEB37	175	2	0.234	0.492	0.524	***	0.437	A	T
SEB38	143	2	0.126	0.500	0.748	***	0.497	A	G
SEB39	140	2	0.036	0.423	0.916	***	0.304	C	T
SEB40	132	2	0.083	0.461	0.819	***	0.360	A	G
SEB41	143	2	0.042	0.332	0.873	***	0.210	C	T
SEB42	135	2	0.289	0.498	0.420	***	0.470	G	T
SEB43	175	2	0.103	0.147	0.301	***	0.080	A	G
SEB44	181	2	0.044	0.084	0.477	***	0.044	A	G
SEB45	178	2	0.315	0.447	0.296	***	0.337	C	T
SEB46	179	2	0.168	0.264	0.365	***	0.156	C	T
SEB47	143	2	0.042	0.067	0.378	***	0.035	A	G

*Note:* Sequence, number of individuals tested (*N*), minor allele frequency (MAF), observed (*H*_o_) and expected (*H*_e_) heterozygosity, inbreeding coefficient (*F*_IS_), and probability for Hardy–Weinberg equilibrium tests (**p < 0.01, ***p < 0.001) are reported. The three diagnostic SNP markers are highlighted in gray.

**FIGURE 2:**
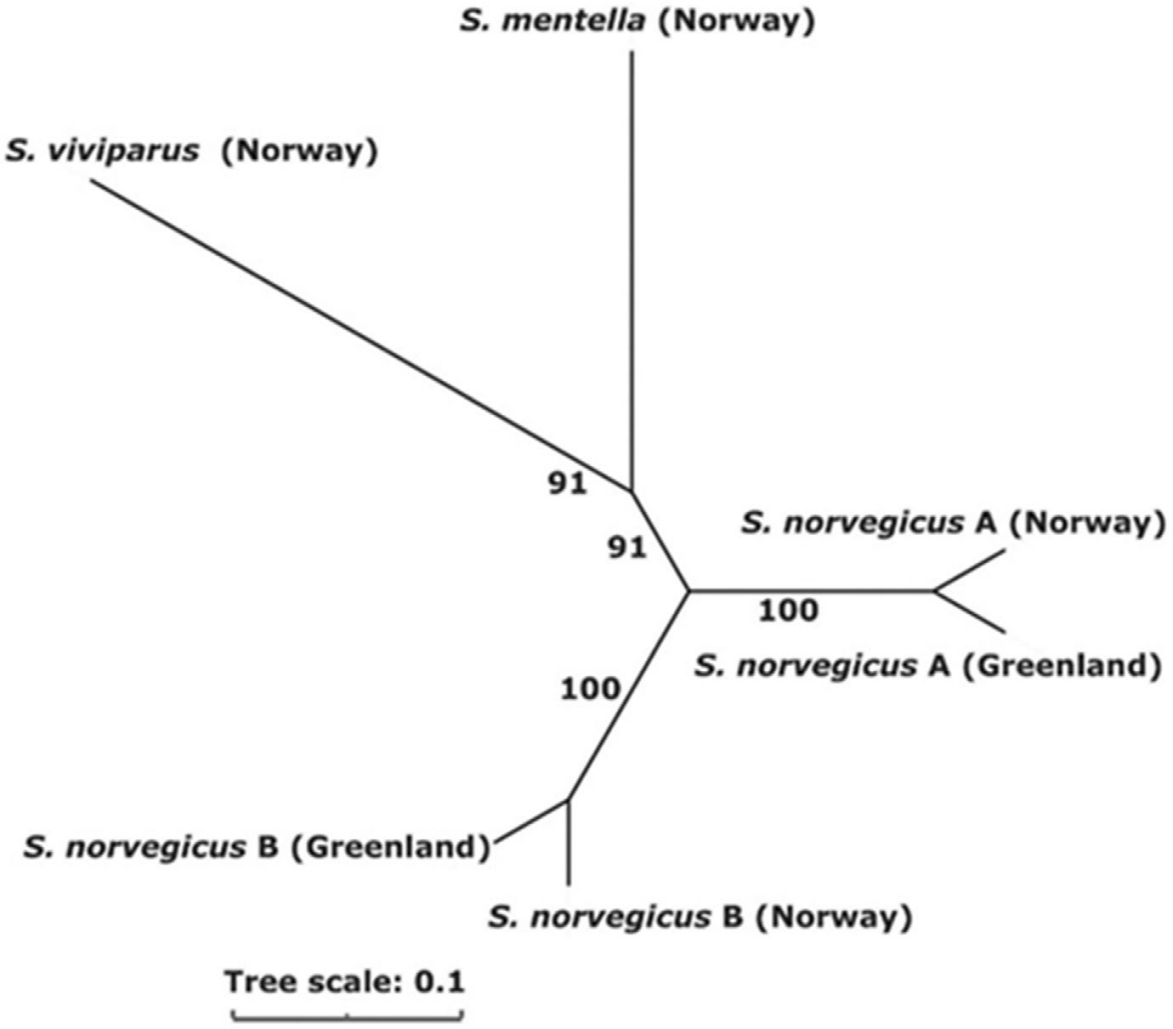
Unrooted NJ tree with the chord distances (*D*_CE_) for the six *Sebastes* clusters obtained through the clustering analysis. The tree is based on data from 49 SNPs genotyped in 191 fish. Bootstrapping values (after 1000 resampling over loci) in percentage, as the degree of support, are presented in the branches.

We show that the *Sebastes* species and *S. norvegicus* types can clearly be discriminated using the 3 or the 47 SNPs based on our results. The separation of these genetic groups, as shown with STRUCTURE, the PCA (Figure 1a,b), or the phylogenetic tree (Figure 2), mirrors that presented in Saha, Hauser, et al. (2017). The present selection of SNPs supports evidence for cryptic species within *S. norvegicus* among both Norwegian and Greenland *Sebastes* as found by microsatellites in Saha, Hauser, et al. (2017) for many of the same specimens. In line with the present findings, Saha, Johansen, et al. (2017) reported that *S. norvegicus* species were more distinct than *S. mentella* and *S. viviparus* .

We selected the three SNPs with fixed alleles in either *S. norvegicus* types, *S. mentella* , or *S. viviparus* , enabling rapid genetic discrimination for monitoring purposes in the Northeast Atlantic (Table 3). The SNP SEB29 and SEB 39 distinguished *S. viviparus* and *S. mentella* from the *S. norvegicus* , while the SEB25 SNP could clearly distinguish between *S. norvegicus* A and *S. norvegicus* B and the other species when combined with the SNP SEB29 and SEB39. The present phylogenetic tree based on 49SNPs (Figure 2) and that from the three SNPs (not shown) showed similar pattern. According to Saha, Johansen, et al. (2017), *S. norvegicus* B is found across the North Atlantic, while *S. norvegicus* A has so far only been identified in the Northeast Atlantic. Even though our initiative to identify these SNP markers was to be able to track the distribution of *S. norvegicus* A and B, we have now a powerful tool for identifying all the Northeast Atlantic species. Most likely are these markers also possible to apply in the Northwest Atlantic where they struggle to discriminate the *Sebastes* sp. There the *S. mentella* and *S. fasciatus* are the most common commercial species and they are landed as “redfish” (Brûlé et al. 2024); however, they do not have genetic marker for possible presence of the *S. norvegicus* (Saha, Hauser, et al. 2017). By collecting DNA samples from *Sebastes* from commercial catches as well as national surveys, this will enable us to, in a fast, effective, and relatively cheap way, to better study the distribution of the *Sebastes* species and monitor the fisheries. In this way, we could monitor the fisheries of *Sebastes* as Norway as we do for the Atlantic cod ( *Gadus morhua* ) during spawning season (Johansen et al. 2018; Dahle et al. 2018).

**TABLE 3:** In total 191 *Sebastes* were analyzed for the three discriminant SNPs.

Species		SEB29 (viv)		SEB39 (ment)		SEB25 (NorA/B)	
*S. mentella*		**C**	**C**	**C**	**C**	**G**	**G**
*S. norvegicus* A		C	C	T	T	**A**	**A**
*S. norvegicus* B		C	C	T	T	G	G
*S. viviparus* *		**T**	**T**	T	T	G	G

*Note:* The bold is meant to higlight the genotype important to identify the species.

* One *S*, *viviparus* showed up as heterozygote individuals in SEB39.

## Author Contributions

**Torild Johansen:** conceptualization (lead), funding acquisition (lead), project administration (lead), writing – original draft (lead). **Tanja Hanebrekke:** data curation (supporting), methodology (supporting), resources (supporting), writing – review and editing (supporting). **Francois Besnier:** formal analysis (supporting), methodology (equal), software (lead), writing – review and editing (supporting). **Jon Ivar Westgaard:** conceptualization (supporting), methodology (supporting), supervision (equal), writing – review and editing (supporting). **Ingrid Marie Bruvold:** methodology (supporting), writing – review and editing (equal). **Geir Dahle:** methodology (supporting), resources (supporting), writing – review and editing (supporting). **Laila Unneland:** data curation (supporting), methodology (supporting), writing – review and editing (supporting). **Helle Torp Christensen:** funding acquisition (supporting), resources (supporting), writing – review and editing (supporting). **Atal Saha:** conceptualization (supporting), formal analysis (supporting), methodology (supporting), software (lead), supervision (equal), visualization (lead), writing – review and editing (supporting).

## Conflicts of Interest

The authors declare no conflicts of interest.

## Supporting information


Data S1.

## Data Availability

Genotypes for all individuals are available as text file online in Supporting Information.
