# Supplementary material for: Development of SNP for Sebastes Species Identification With Special Focus on the Cryptic Species Complex of Sebastes norvegicus
Source: Ecol Evol. 2025 Jan 8;15(1):e70767. doi: 10.1002/ece3.70767 (PMC11711104; doi:10.1002/ece3.70767)
Supplement: Supplementary file 1 — Data S1. [file ECE3-15-e70767-s001.zip › Suplement SNP note 5nov.docx]

Supplement information

Figure S1. PCA plot of the sequenced *Sebastes* species based on the total of 10750 SNPs (quality filtered .vcf file , i.e the output from SNP calling). 486 *Sebastes* specimens from previous projects (e.g. Saha et al., 2017, 2021): 174 *Sebastes norvegicus*, 277 *S. mentella,* 35 *S. viviparus* specimens were collected and morphologically identified from eight locations across the North Atlantic


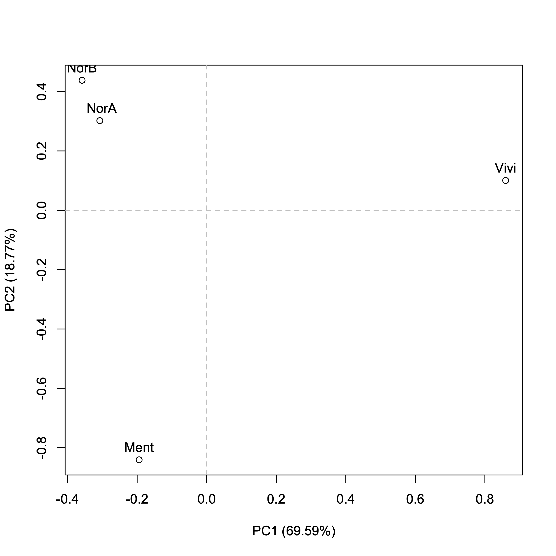

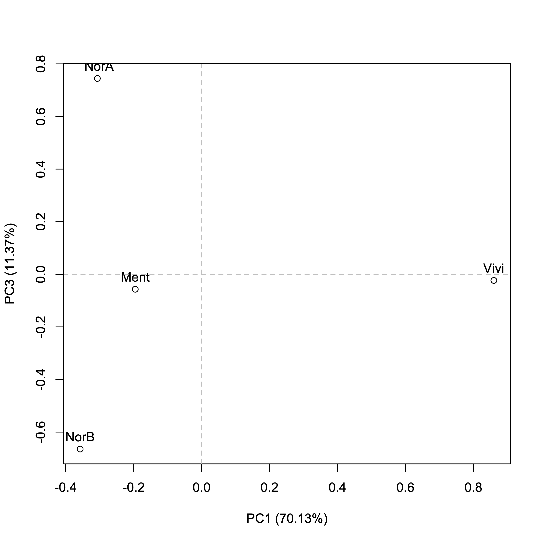


Table S1. Primer assay for the three SNP markers for species identification of the Northeast Atlantic *Sebastes*.

| Assay | Primers and probe | Sequence | 5' Dye |
| --- | --- | --- | --- |
| SEB29 | NorA-Viv-169_Fwd | GTGGTCAACAGATGAGAAACAGACT |  |
|  | NorA-Viv-169_Rev | ACCAATTATGTCACTGACAATATACTGTACATATTTTT |  |
|  | NorA-Viv-169_Probe1 | TTGTTTGCTGCTCATTAGA | VIC |
|  | NorA-Viv-169_Probe2 | TTTGCTGCCCATTAGA | FAM |
| SEB39 | NorBMent230_Fwd | TGGTCAGGTTCAATATTACAATGAATGCT |  |
|  | NorBMent230_Rev | TGCTGACAGAGGAAATCATCTTGTT |  |
|  | NorBMent230_Probe1 | ACCATCAGTTTGTCATAAC | VIC |
|  | NorBMent230_Probe2 | CCATCAGTTTGCCATAAC | FAM |
| SEB25 | NorA-NorB-138_Fwd | GCTGATTCGCCATTCATGACA |  |
|  | NorA-NorB-138_Rev | TCTGACGTCATAACTACCTGAAGTCT |  |
|  | NorA-NorB-138_Probe1 | CAAGCATCCGTGAGGAG | VIC |
|  | NorA-NorB-138_Probe2 | CAAGCATCCATGAGGAG | FAM |
